# Current Unveiling Key Research Trends in Endometrial Cancer: A Comprehensive Topic Modeling Analysis

**DOI:** 10.3390/healthcare13131567

**Published:** 2025-06-30

**Authors:** Sujin Kang, Youngji Kim

**Affiliations:** 1 College of Nursing, The Catholic University of Korea, Seoul 06591, Republic of Korea; 2 Department of Nursing, College of Nursing and Health, Kongju National University, Gongju-si 32588, Republic of Korea

**Keywords:** endometrial cancer, social network analysis

## Abstract

**Background/Objectives:** Endometrial cancer (EC) is the sixth most common cancer among women worldwide, and its global incidence has significantly increased over the past three decades. Despite its substantial burden, comprehensive reviews of EC-related research remain limited. This study employs topic modeling to analyze and classify recent research trends in EC. **Methods:** We identified studies related to endometrial carcinoma published between 2019 and 2023 in PubMed, Web of Science, and the Cochrane Library. The search was conducted using the following terms: endometr* AND (neoplasm* OR cancer* OR carcinoma*) NOT endometriosis. Word clouds were constructed and topic modeling was performed to analyze research activity. **Results:** A total of 2188 studies were selected, and 11,552 terms were extracted. High-frequency and TF-IDF-weighted keywords included ‘cancer’, ‘risk’, ‘survival’, ‘stage’, ‘tumor’, ‘surgery’, and ‘OS.’ Topic modeling analysis identified ten clusters, categorized as follows: ‘Gynecologic cancer’, ‘Surgical staging’, ‘Therapeutic efficacy’, ‘Diagnosis’, ‘Surgical management’, ‘Multimodal treatment’, ‘Molecular treatment’, ‘Risk factors’, ‘Survival’, and ‘Hormonal regulation.’ **Conclusions:** This study highlights that recent research on EC has primarily focused on surgical decision making, outcome prediction, and patient survival. Future studies should place greater emphasis on multimodal treatment and prevention—particularly through the identification of risk factors—as well as on improving patients’ quality of life.

## 1. Introduction

Endometrial cancer (EC) is the sixth most common cancer among women worldwide [1]. Its global incidence has increased significantly, rising from 187,191 cases in 1990 to 435,041 in 2010, highlighting the urgent need to understand and address this disease [2]. Obesity, advanced age, and low-grade inflammation have been linked to an increased risk of ED. These associations require further investigation to validate the findings across diverse groups of women in a broader population [3,4].

Despite its substantial global burden, the research landscape of EC has not been comprehensively reviewed, leaving gaps in our understanding of the breadth and focus of current scientific investigations [5].

While many aspects of EC—including risk factors, treatment modalities, and patient outcomes—are well established, ongoing advances in areas such as prevention, personalized medicine, and survivorship care continue to refine our understanding and management of this disease [6]. Consequently, a comprehensive and updated review of the current literature is warranted to map recent research trends, highlight emerging areas, and identify knowledge gaps for which further research may enhance patient outcomes. Previous reviews have typically concentrated on specific facets of the disease, such as clinical interventions or epidemiological studies, rather than providing a holistic view of the research activities [7,8,9].

This review aims to provide a comprehensive synthesis of current research to guide future studies, deepen insights into EC, and support the development of more effective, holistic care strategies for women with this disease.

## 2. Materials and Methods

### 2.1. Study Design and Data Sources

This study is a quantitative content analysis that applied text network analysis and topic modeling to literature published over the past five years to identify research trends in EC. Data were collected from PubMed, Web of Science, and the Cochrane Library, focusing on publications specific to EC.

### 2.2. Literature Search

We identified research related to endometrial carcinoma published in the aforementioned databases between 2019 and 2023. Given the extensive volume of global research on EC, we selected a recent five-year period to effectively identify emerging research patterns and clarify the focus of our analysis. The search was conducted in Aug 2023 using the following terms in the abstract field: endometr* AND (neoplasm* or cancer* or carcinoma*) NOT endometriosis. Searching within abstracts was intended to enhance the validity of the search and ensure a thorough review of literature directly relevant to EC. We limited our search to “original articles” by applying the “document type” filter, thereby excluding reviews, news items, and other non-original publications not aligned with our research objective.

**Figure 1:**
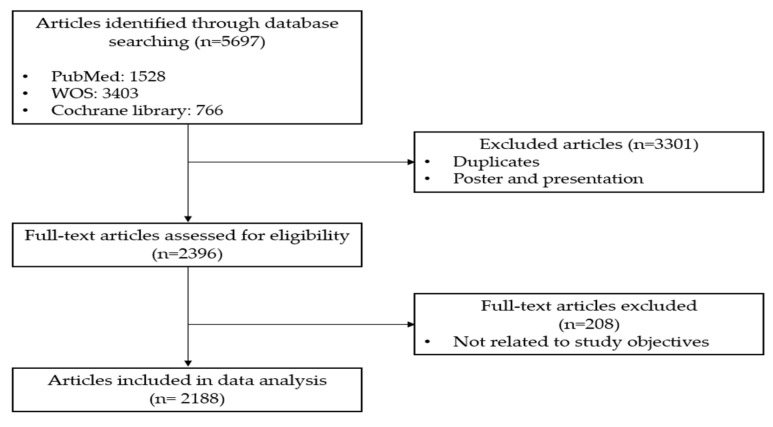
PRISMA (Preferred Reporting Items for Systematic Reviews and Meta-Analyses) flow diagram illustrating the process of article selection.

The initial search retrieved a total of 5697 articles (PubMed: 1528 articles, Web of Science: 3403 articles, and Cochrane Library: 766 articles) between 2019 and 2023. To ensure the uniqueness of the articles, duplicate entries were identified and removed using EndNote 20.6 (Thomson Reuters, Philadelphia, PA, USA). This process resulted in the exclusion of 3301 duplicate articles. Following the removal of duplicates, 2396 articles were retained for the first round of data analysis. These articles were independently screened for relevance based on their titles and abstracts by two researchers and an external gynecologist, according to predefined inclusion and exclusion criteria. During this process, 208 articles were excluded for not aligning with the study objectives. Discrepancies were resolved through discussion among the review team. Consequently, 2188 articles were included in the final dataset for topic modeling (Figure 1).

### 2.3. Data Analysis and Categorization

Text network analysis and topic modeling was performed to examine research activity. A total of 2188 abstracts were selected. Using NetMiner (https://www.netminer.com), only nouns were extracted as keywords from the abstract, and terms were extracted for data analysis using dictionary (including negative words, designated words, and a thesaurus). Thesaurus terms include the following: SLN (sentinel lymph node), EC (endometrial cancer), LVSI (lymphovascular space invasion), OS (overall survival), and PCOS (polycystic ovary syndrome). Common general terms (e.g., patient, study, woman, treatment, result, group, CI, and rate) were excluded as stop words. Negative words include terms such as not, no, never, none, fail, lack, impossible, inadequate, poor, and insufficient.

Data were cleaned to obtain meaningful morphemes. Two external experts (including an obstetrician and gynecologist) and a researcher developed a list of synonyms, designated words, and exclusion words based on their expertise in the topic of EC research and refined keywords. A synonym dictionary was constructed to unify similar terms (e.g., ‘obese’ and ‘obesity’ were merged as ‘obesity’). Designated words are a collection of phrases that are treated as a single unit because multiple words convey a single meaning. For example, lymphovascular space invasion is recognized as LVSI. The exclusion list included research-related terms (purpose, method, result, and conclusion) to be excluded from the analysis.

As a result, a total of 24,703 semantic morphemes (keywords) were extracted. A word cloud was generated by selecting only words that appeared more than four times in the papers. Centrality and coherence analyses were performed using only terms that had three or more linkages in the network, resulting in the extraction of 11,552 terms.

A word cloud is a visual representation of text data, in which the size of each word indicates its frequency or importance in the dataset [10]. In this study, word clouds by frequency and TF-IDF were used to visualize the most common terms in the abstracts of publications related to EC research.

Topic modeling is a probabilistic model that calculates and discovers major topics for each document [11]. In this study, the Latent Dirichlet Allocation (LDA) algorithm was applied to identify the latent meaning in the text. LDA is a widely used topic modeling technique in natural language processing and machine learning that identifies abstract topics within a collection of documents [12]. It works under the assumption that each document is a mixture of various topics and that each word within the document is attributable to one of the document’s topics [12]. The topic model was trained using the MCMC method with an alpha value of 2.0, a beta value of 0.1, and an iteration count of 1000.

Lda2vec is an unsupervised text mining method, and determining the optimal number of topics is crucial [13]. There is no definitive method for selecting the optimal number of topics [14]. The perplexity measure can estimate the optimal number, but its results can be challenging to interpret [14]. Typically, researchers decide on the optimal number of topics. In our study, we tested Lda2vec with 10, 15, and 20 topics, comparing the similarity and differences in topic content across these models to determine the optimal number. While reading the keywords and abstracts included in each cluster, the researchers discussed and named the number and names of the subject groups. Although both LDA and Lda2vec were explored during the modeling phase, the final analysis and topic interpretation were conducted using the LDA model due to its better coherence scores and greater interpretability.

## 3. Results

**Figure 2:**
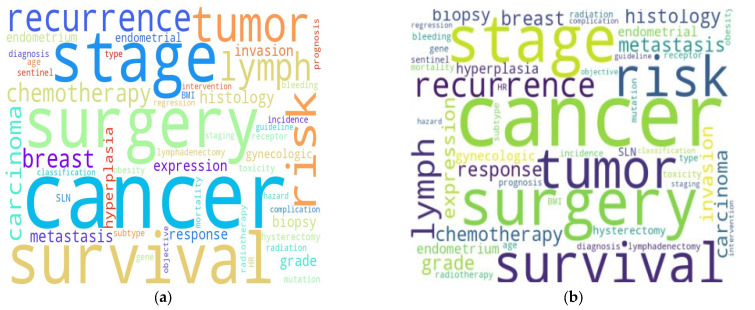
Comparative word cloud visualizations highlighting term relevance: (**a**) frequency-based and (**b**) TF-IDF-weighted.

We generated word clouds based on different criteria to visualize key terms within the dataset. The first word cloud reflects the term frequency, highlighting the most commonly occurring words. The second is based on TF-IDF (term frequency–inverse document frequency), emphasizing terms that are particularly important in specific documents but less frequent across the entire corpus. Both visualizations consistently highlight key terms—such as “cancer”, “risk”, “surgery”, “tumor”, “survival”, and “stage”—indicating that these are significant keywords within the dataset (Figure 2).

The topic modeling analysis on endometrial neoplasms has identified ten key research areas, each characterized by specific keywords. The ten identified topics are as follows: ‘gynecologic cancer’, ‘surgical staging’, ‘therapeutic efficacy’, ‘diagnosis’, ‘surgical management’, ‘multimodal treatment’, ‘molecular treatment’, ‘risk factors’, ‘survival’, and ‘hormonal regulation’. The most probable keywords for each topic are provided in Table 1.

**Figure 3:**
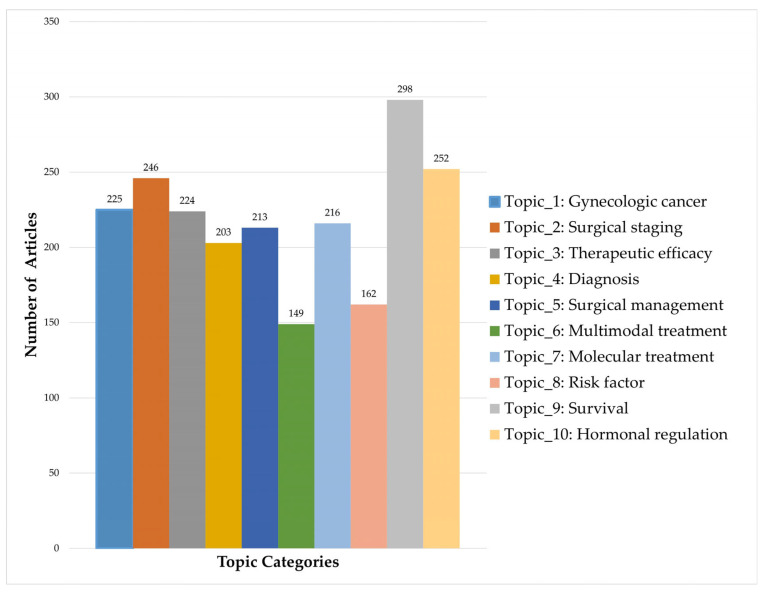
Literature volume by research topic.

‘Survival’ emerged as the most frequently studied topic, while ‘multimodal treatment’ and ‘risk factors’ were notably underrepresented. The remaining topics appeared with relatively similar frequencies (Figure 3).

## 4. Discussion

This study employed topic modeling to analyze 2188 original research articles on endometrial neoplasms, identifying ten distinct thematic areas. These topics highlight key areas of clinical and scientific interest in gynecologic oncology, offering insights into contemporary research directions. Together, they capture a comprehensive view of the evolving priorities in EC research and its broader implications for women’s health.

To contextualize thematic priorities within the endometrial neoplasm literature, we employed word cloud visualizations based on both term frequency and TF-IDF scoring. Across both methods, commonly emphasized terms included “cancer”, “risk”, “surgery”, “tumor”, “survival”, and “stage”, reflecting consistent focus areas in gynecologic oncology. These terms reflect core aspects of clinical management and emphasize continued focus on prognosis, surgical decision making, and outcome prediction in EC.

While term frequency captures general lexical prominence, the TF-IDF approach enabled the identification of contextually significant terms that may not be frequent overall but are highly informative within specific documents. The convergence of results between these two approaches reinforces the centrality of oncologic risk assessment and surgical intervention in the literature. Clinically, this trend underscores continued efforts to enhance precision staging, optimize risk-adapted therapies, and better understand survival determinants.

While surgical strategies and outcome prediction remain central to EC management, emerging evidence highlights the growing need for comprehensive research and the implementation of multimodal treatment approaches—including surgery, chemotherapy, radiotherapy, and immunotherapy—to improve outcomes across all patient groups [35,36].

As a result of topic modeling in this study, publications related to “survival” were the most prevalent, indicating increasing interest in patient survival rates and associated prognostic factors. In particular, “overall survival (OS)” is widely regarded as a key prognostic indicator, and recent studies have extensively examined how variables such as “stage”, “grade”, and “recurrence” risk influence OS. Among these, “stage” and “grade” are established as critical predictors of OS [37], while the depth of myometrial “invasion” is an important factor for assessing recurrence risk [38]. These trends underscore the importance of accurate staging, the confirmation of tumor grade and invasion depth, and the development of individualized treatment strategies to improve survival outcomes in EC patients. The “gynecologic cancer” topic reflects the integration of EC research with broader oncology studies, including those on breast, ovarian, and lung cancers. The inclusion of keywords such as “breast”, “prostate”, “OC (ovarian cancer)”, and “lung” indicates that EC research is increasingly being integrated with studies on other major malignancies. This indicates a growing emphasis on integrated approaches in oncology, with increasing interest in the interactions among different cancer types and shared risk factors [39,40]. The presence of keywords like “gene” suggests increasing interest in the genetic and molecular mechanisms of disease, which supports the development of targeted therapies and refined disease classifications [41].

“Surgical staging” emerged as a major topic, highlighting the critical importance of metastasis evaluation and “lymph” node status in determining prognosis and guiding treatment decisions for EC patients. Approximately 10% of patients initially believed to have cancer confined to the uterus were later found to have lymph node metastasis upon surgical examination [42]. The prominence of keywords such as “lymphadenectomy” and “SLN (sentinel lymph node)” reflects active research on surgical techniques and diagnostic procedures in EC. SLN “biopsy” has improved the detection of lymph node metastasis while reducing false-negative results [43]. These findings underscore ongoing efforts to develop more effective and accurate methods for evaluating metastatic spread in EC.

“Therapeutic efficacy” reflects the increasing interest in the effectiveness and safety of emerging treatments. In the management of EC, “efficacy” is a key metric for evaluating treatment “response” and survival outcomes. Recent clinical trials have demonstrated that the “combination” of immunotherapy and targeted therapy yields higher response rates compared to conventional chemotherapy [44]. When introducing novel treatments, “toxicity” and “safety” are critical considerations. These findings highlight the importance of balancing therapeutic efficacy with adverse effects and underscore the need to develop optimal treatment strategies that maximize patient benefit while minimizing harm.

The “diagnosis” of EC plays a critical role in determining patient prognosis and guiding treatment planning. The prominence of “diagnosis” as a major topic in this study highlights the importance of early detection and advancements in diagnostic techniques. The most common initial symptom of EC is “bleeding,” with abnormal postmenopausal vaginal bleeding being considered a significant “symptom” that requires immediate medical evaluation [45]. Various methods are used to evaluate the “uterine” endometrium during the diagnostic process. Transvaginal ultrasound is commonly employed as an initial test to assess thickness and detect abnormalities of the “endometrium,” while endometrial biopsy is regarded as the most accurate method for diagnosing “malignancy” [46]. Additionally, hysteroscopy allows for the direct visualization of endometrial lesions, aiding in the differentiation between “polyps” and other benign “lesions” [46]. These findings reinforce the critical role of early detection and precise staging in effective EC management. Furthermore, recent advances in molecular diagnostics and non-invasive diagnostic techniques are expected to enhance diagnostic accuracy and ultimately improve patient outcomes.

The emergence of “surgical management” reflects the significance of surgical approaches in the treatment of EC. “Hysterectomy” remains one of the most commonly performed surgical procedures for managing various gynecological conditions. Recent studies comparing “laparotomy” and “laparoscopy” have demonstrated that laparoscopic surgery leads to a faster recovery and fewer complications than open surgery [47]. Factors such as “BMI,” “weight,” and “age” significantly affect surgical outcomes [48]. In particular, surgery is more complex in obese patients, with an increased risk of complications [48], highlighting the need for a tailored approaches in this population. Postoperative “complication” management is critical, as it directly influences patient quality of life. These research trends reaffirm the importance of surgery in the EC treatment and underscore the need for personalized strategies tailored to each patient.

The emergence of “molecular treatment” as a topic underscores the growing importance of molecular profiling in understanding EC pathogenesis and informing targeted therapies. According to the Cancer Genome Atlas (TCGA), EC can be divided into four molecular “subtypes”: POLE ultramutated, microsatellite instability (MMRd), copy-number low (NSMP), and TP53 mutated [49]. This “classification” provides greater “specificity” and “sensitivity” than traditional histological classifications, improving both prognosis prediction and treatment planning [49]. In addition, gene mutations related to DNA “repair” mechanisms have become a key area of research. Recent studies suggest that combining PARP inhibitors with immunotherapy may be effective in treating specific molecular subtypes [50]. Molecular biological approaches continue to play a vital role in advancing personalized treatment strategies for EC [51].

The emergence of “hormonal regulation” highlights the significant influence of hormonal factors and fertility preservation on the development, progression, and treatment of EC. The intersection between pregnancy and endometrial tumors represents a complex area of research, with keywords such as “hyperplasia,” “fertility,” and “hormone” reflecting growing concerns about the impact of cancer and its treatment on reproductive health. Effectively managing the tumor while preserving fertility remains a major challenge, requiring a multidisciplinary approach [52]. In particular, “progestin” therapy has gained attention as a conservative treatment option for young EC patients and those with precancerous lesions, offering an important alternative for patients seeking fertility preservation [53]. These research trends indicate that the understanding of EC is expanding beyond traditional oncological perspectives to include aspects of reproductive endocrinology.

This study found that publications on “multimodal treatment” and “risk factor” were relatively limited. “Multimodal treatment”, which involves the use of multiple therapeutic approaches such as chemotherapy and radiation therapy [54], is essential; however, the lack of research in this area suggests that clinicians may be without sufficient evidence-based guidelines for making optimal treatment decisions. The introduction of immunotherapy and targeted therapy has further complicated the process of identifying the most effective combinations with existing chemotherapy and radiation therapy. For example, although the combination of pembrolizumab and lenvatinib has demonstrated effectiveness, research on various combinations of these newer agents with conventional treatments remains insufficient [44]. This knowledge gap may pose challenges for clinicians in determining the most appropriate sequence and combination of treatments for individual patients. Based on the findings of this study, the limited representation of immunotherapy and targeted therapy is acknowledged as a limitation. Future research should aim to fill this gap to enable a more comprehensive and evidence-based approach to treatment.

The lack of research on “risk factor” identified in this study carries significant clinical implications. A comprehensive understanding of the risk factors associated with EC remains limited, which may impede the development of effective prevention and early detection. This study yielded results consistent with those of the 2016 gap analysis [55], which identified the identification of high-risk groups for screening and prevention as the top research priority in EC based on input from patients, clinicians, and the general public.

Overall, among the ten topics derived in this study, only one was related to prevention, and it was limited to risk factors, indicating that more active research in the area of prevention is needed.

According to the statistics from the Health Insurance Review & Assessment Service, the number of patients receiving medical treatment for EC increased from 7505 in 2010 to 22,088 in 2023—an approximate increase of 194% [56]. Notably, the number of young patients in their 20s and 30s has also shown a marked rise [56]. The rising incidence of EC among younger women is attributed to several contributing factors, including the increasing number of women who do not undergo pregnancy or childbirth [57], the earlier onset of menarche [58], and the growing prevalence of obesity associated with a more Westernized diet [59]. Another suspected factor is increased exposure to endocrine-disrupting chemicals [60]. These chemicals share a structural similarity with estrogen, which may lead the reproductive system to misinterpret them, resulting in endocrine system disruption [60]. “Obesity” has been identified as the most significant risk factor for EC [61]. Studies have shown that as body mass index (BMI) increases, both the “incidence” and “mortality” of EC rise substantially [61]. Specifically, for every five kg/m^2^ increase in BMI, the risk of developing EC increases by 50% [62]. “Age” is another critical risk factor for the disease [36]. Most cases of EC occur in postmenopausal women [63], and “population”-based studies consistently show that risk increases with age [64].

During the COVID-19 pandemic, people were required to stay home and reduce activities such as dining out, which led to a significant increase in the consumption of delivered meals [65]. These meals were often served in hot plastic food containers, thereby increasing exposure to endocrine-disrupting chemicals [66]. This heightened exposure is believed to have contributed to the rising incidence of EC among young women. In light of this recent increase, a more comprehensive understanding of “risk” factors—such as lifestyle behaviors, obesity, and related environmental change —is urgently needed.

Moreover, research on innovative therapies, including immunotherapy and targeted molecular treatments, needs to be expanded to better understand their effectiveness in managing endometrial neoplasms [67,68]. There are no screening programs available or recommended for the early detection of EC, so diagnosis relies on recognizing and investigating suspicious symptoms [63]. Future research should explore the feasibility of preventive approaches, particularly in high-risk populations. Lastly, greater attention should be given to research on patient education and awareness, especially regarding their roles in early detection, treatment adherence, and disease management. Addressing these research gaps will contribute to a more holistic understanding of endometrial neoplasms and ultimately lead to improved patient care and outcomes. EC remains an under-researched field despite its high disease burden, and more vigorous research efforts are necessary to extend patient survival and improve the quality of life of survivors.

Through a topic modeling analysis of research trends in EC, it was revealed that topics such as prevention, risk factor identification, immunotherapy, and multimodal treatment were inadequately represented in the current literature. This gap likely comes from several contributing factors. First, research funding in gynecologic oncology tends to prioritize treatment-focused studies over preventive or emerging approaches, resulting in fewer resources allocated to innovation in these areas [69]. Second, immunotherapy is still a relatively recent addition to endometrial cancer treatment, and thus remains in the early stages of widespread clinical adoption and investigation [67,68]. Additionally, the underrepresentation of these themes may reflect a broader lack of multidisciplinary collaboration in the field. Addressing prevention and immunotherapy requires integrated expertise from oncology, immunology, molecular biology, public health, and health policy fields. However, current research frameworks often remain compartmentalized within single disciplines, slowing the translation of novel approaches into clinical practice [70]. Recognizing these structural, scientific, and organizational limitations is essential for shaping future research agendas and guiding strategic investment in underexplored but high-potential areas of endometrial cancer research.

This study offers several meaningful contributions to the field of gynecologic oncology. While our study may not serve as a clinical guideline, its aim is to provide a macroscopic view of research trends through topic modeling—a perspective valuable to researchers new to the field or those interested in bibliometric insights. It provides an up-to-date summary of over 2000 recent articles on EC, offering a broad view of current research trends. We used topic modeling and text mining, which are innovative tools not commonly applied in this field. We identified under-researched areas like prevention and combination therapy, helping guide future studies. This study found that studies on patient quality of life are limited, particularly in relation to how endometrial neoplasms and their treatment affect patients’ physical, emotional, and social well-being. There is also a notable lack of research on long-term outcomes and follow-up care for patients who have undergone treatment for endometrial neoplasms. Disparities in healthcare—such as socioeconomic, racial, and geographic differences in access to treatment and outcomes—likewise warrant greater attention. Our findings can also help non-oncology experts, such as public health and data science professionals, understand the current research landscape.

One of the limitations of this study is that it did not differentiate between histologic subtypes of EC, such as endometrioid, serous, clear cell, undifferentiated, dedifferentiated, mixed carcinoma, and carcinosarcoma subtypes. These subtypes have distinct molecular features, clinical behaviors, and prognoses. Future studies should explore research trends within each histologic subtype to provide more granular insights and inform subtype-specific clinical strategies. Another limitation is that in topic modeling analysis, the selection of the number of topics is inherently subjective, and the way words are grouped may lead to variations in results depending on the researcher. In this study, all analytical procedures were described in detail accordingly, and such considerations should be further emphasized in future research.

## 5. Conclusions

This study provides a comprehensive overview of recent research trends in EC by employing topic modeling, thereby offering researchers a valuable roadmap for cancer prevention and future investigations. Our findings indicate that the current body of literature predominantly focuses on patient survival, surgical strategies, and outcome prediction. However, there remains a critical need to expand research efforts toward multimodal treatment, the identification of risk factors, and the development of effective prevention strategies. Such efforts will not only deepen our understanding of endometrial neoplasms but also facilitate improved clinical management and patient care.

Furthermore, we emphasize the importance of redirecting part of the research focus toward prevention—including the identification and modification of risk factors—and initiatives aimed at enhancing patients’ quality of life. Broadening the research scope in these directions will enable future studies to contribute more substantially to reducing the incidence of EC and improving long-term outcomes. In addition, we strongly encourage future research to place greater emphasis on long-term outcomes and follow-up, as these aspects are essential for optimizing survivorship and the overall quality of care.

## Figures and Tables

**Table 1:** Topic groups in endometrial cancer studies.

Topic Name	Keyword										Related References
	1	2	3	4	5	6	7	8	9	10	
Gynecologic cancer	cancer	breast	endometriosis	survivor	gene	RR	heterogeneity	prostate	OC	lung	Li et al., 2022 [15] Lv et al., 2022 [16]
Surgical staging	lymph	SLN	metastasis	lymphadenectomy	biopsy	staging	ICG	dissection	laparoscopic	sensitivity	Matanes et al., 2022 [17] Cabezas Palacios et al., 2022 [18]
Therapeutic efficacy	response	combination	activity	efficacy	event	toxicity	safety	inhibitor	death	chemotherapy	Singh et al., 2022 [19] Yang et al., 2019 [20]
Diagnosis	diagnosis	uterine	malignancy	bleeding	syndrome	endometrium	symptom	lesion	polyp	gene	Aksel & Çakir, 2020 [21] Bi et al., 2020 [22]
Surgical management	surgery	hysterectomy	complication	BMI	score	blood	weight	age	laparotomy	laparoscopy	Odetto et al., 2023 [23] Saini et al., 2023 [24]
Multimodal treatment	chemotherapy	radiotherapy	radiation	brachytherapy	FIGO	RT	imaging	MRI	care	cost	Emons & Vordermark, 2019 [25] Egawa-Takata et al., 2022 [26]
Molecular treatment	tumor	carcinoma	mutation	classification	subtype	specificity	subgroup	sensitivity	PFS	repair	Talhouk et al., 2023 [27] Watanabe et al., 2023 [28]
Risk factor	risk	incidence	obesity	mortality	age	population	prevalence	RR	mass	LS	Gao et al., 2022 [29] Koual et al., 2018 [30]
Survival	survival	OS	stage	recurrence	grade	invasion	histology	HR	LVSI	type	Wilson et al., 2023 [31] Cusano et al., 2018 [32]
Hormonal regulation	hyperplasia	expression	tumor	receptor	cytology	pregnancy	hormone	fertility	progestin	estrogen	Pal et al., 2018 [33] Tamauchi et al., 2018 [34]

RR relative risk; OC Oral contraceptive; SLN Sentinel lymph node; ICG indocyanine-green; FIGO International Federation of Gynecology and Obstetrics; RT Radiation therapy; MRI Magnetic resonance imaging; EC Endometrial cancer; PFS Progression-free survival; LS Left salpingectomy; OS Overall survival; HR Hazard risk; LVSI Lymphovascular space invasion.

## Data Availability

The data presented in this study are available on request from the corresponding author.
